# ProBDNF and Brain-Derived Neurotrophic Factor Prodomain Differently Modulate Acetylcholine Release in Regenerating and Mature Mouse Motor Synapses

**DOI:** 10.3389/fncel.2022.866802

**Published:** 2022-05-03

**Authors:** Polina O. Bogacheva, Anastasia I. Molchanova, Ekaterina S. Pravdivceva, Anna S. Miteva, Olga P. Balezina, Alexander E. Gaydukov

**Affiliations:** Faculty of Biology, Department of Human and Animal Physiology, Lomonosov Moscow State University, Moscow, Russia

**Keywords:** neuromuscular synapse, acetylcholine, proBDNF, BDNF prodomain, GIRK, p75, ROCK, pannexin 1 (Panx1)

## Abstract

The effects of brain-derived neurotrophic factor (BDNF) processing by-products (proBDNF and BDNF prodomain) on the activity of mouse neuromuscular junctions (NMJs) were studied in synapses formed during the reinnervation of extensor digitorum longus muscle (m. EDL) and mature synapses of the diaphragm. The parameters of spontaneous miniature endplate potentials (MEPPs) and evoked endplate potentials (EPPs) were analyzed in presence of each of the BDNF maturation products (both – 1 nM). In newly formed NMJs, proBDNF caused an increase in the resting membrane potential of muscle fibers and a decrease in the frequency of MEPPs, which was prevented by tertiapin-Q, a G-protein-coupled inwardly rectifying potassium channels (GIRK) blocker but not by p75 receptor signaling inhibitor TAT-Pep5. proBDNF had no effect on the parameters of EPPs. BDNF prodomain in newly formed synapses had effects different from those of proBDNF: it increased the amplitude of MEPPs, which was prevented by vesamicol, an inhibitor of vesicular acetylcholine (ACh) transporter; and reduced the quantal content of EPPs. In mature NMJs, proBDNF did not influence MEPPs parameters, but BDNF prodomain suppressed both spontaneous and evoked ACh release: decreased the frequency and amplitude of MEPPs, and the amplitude and quantal content of EPPs. This effect of the BDNF prodomain was prevented by blocking GIRK channels, by TAT-Pep5 or by Rho-associated protein kinase (ROCK) inhibitor Y-27632. At the same time, the BDNF prodomain did not show any inhibitory effects in diaphragm motor synapses of pannexin 1 knockout mice, which have impaired purinergic regulation of neuromuscular transmission. The data obtained suggest that there is a previously unknown mechanism for the acute suppression of spontaneous and evoked ACh release in mature motor synapses, which involves the activation of p75 receptors, ROCK and GIRK channels by BDNF prodomain and requires interaction with metabotropic purinoreceptors. In general, our results show that both the precursor of BDNF and the product of its maturation have predominantly inhibitory effects on spontaneous and evoked ACh release in newly formed or functionally mature neuromuscular junctions, which are mainly opposite to the effects of BDNF. The inhibitory influences of both proteins related to brain neurotrophin are mediated via GIRK channels of mouse NMJs.

## Introduction

Brain-derived neurotrophic factor (BDNF) is well known as a regulator of neuronal development and differentiation and as a modulator of neurotransmission in CNS synapses (Park and Poo, 2012; Castrén and Antila, 2017; Wang et al., 2022). The presence of both BDNF and its receptors has been shown in all three compartments of neuromuscular junctions (NMJs) – axon terminals, perisynaptic Schwann cells, and muscle fibers (Garcia et al., 2010b). Moreover, nowadays BDNF is considered a myokine as it can be released from the muscle and act on nerve terminals as a retrograde messenger (Hurtado et al., 2017; Gaydukov et al., 2019). Recently, it became obvious that the numerous synaptic effects of BDNF cannot be unambiguously interpreted without taking into account the possible simultaneous action of its maturation by-products.

Like other neurotrophins, BDNF is initially synthesized as a protein precursor pre-proBDNF (consisting of 247 amino acid residues in humans) (Costa et al., 2017). After its pre-sequence is cleaved off, proBDNF (∼32 kDa) is formed, which is known to have a signaling function (Lu, 2003; Matsumoto et al., 2008). ProBDNF, in turn, is processed into mature BDNF (∼13 kDa) and a residual propeptide, or BDNF prodomain. Cleavage of proBDNF may occur intracellularly, in the *trans-*Golgi network by furin or in secretory vesicles by PC-convertases (Wang et al., 2021), and extracellularly, after the release of proBDNF into the synaptic cleft, by matrix metalloproteinases (Mizoguchi et al., 2011) or tPA/plasmin system (Pang et al., 2004; Gray and Ellis, 2008; Nagappan et al., 2009).

The evidence of receptor-specific action of proBDNF as an independent signaling molecule secreted along with BDNF have been described in CNS synapses (Koshimizu et al., 2009; Costa et al., 2017; De Vincenti et al., 2019). Much less is known about the possible signaling role of the BDNF prodomain. A few recent data suggest that BDNF prodomain, like proBDNF, may play an independent role in CNS synapses, and both these processing by-products often act as functional antagonists of BDNF in the regulation of neurotransmitter release and synaptic plasticity (Mizui et al., 2016; Kojima et al., 2019).

Expression of proBDNF and its myogenic release along with BDNF has also been described in mouse NMJs during neonatal development of synaptic contacts with skeletal muscle fibers. Notably, these peptides had opposite effects on ACh release and stabilization of synapses due to activation of different presynaptic receptors – TrkB for BDNF and p75 for proBDNF (Je et al., 2012, 2013). An acute increase in the level of proBDNF and p75 receptors expression was recently revealed in mature mouse skeletal muscle in response to its denervation (Aby et al., 2021). However, the role of this upregulation of proBDNF and the range of its retrograde effects during the period of muscle reinnervation and synaptic formation were not studied yet.

In contrast to that, we have previously shown that mature BDNF potentiates ACh release both in newly formed and mature motor synapses (Gaydukov et al., 2018, 2019).

Along with the under-investigated activity of proBDNF, nothing is known about the possible effects of BDNF prodomain in regenerating NMJs after nerve injury and functionally mature motor synapses.

In this regard, the aim of this study was to reveal and compare the effects of two BDNF processing byproducts – proBDNF and BDNF prodomain – on quantal acetylcholine (ACh) release and to provide insights into mechanisms underlying these effects in newly formed and mature mouse neuromuscular junctions.

## Materials and Methods

### Animals and Surgery

Data presented were obtained from isolated neuromuscular preparations of extensor digitorum longus muscle (*m. EDL*) and hemidiaphragm of adult mice (C57Bl/6 strain, 25–30 g body weight) of either sex. The strain of pannexin 1 knockout mice (Panx1^–/–^) was initially generated from C57Bl/6 by V.I. Shestopalov and characterized previously (Dvoriantchikova et al., 2012). Animals were housed in groups of 4–6 mice/big cage with enriched environment and kept at a constant temperature (22°C) and regular (12/12 h) light–dark cycle. Food and water were provided *ad libitum*. All the procedures were performed in accordance with the EC guidelines (Directive 86/609/EEC on the treatment of laboratory animals). The protocol of experimental procedures (N° 2021-10-21-11.3SOD) was approved by the Bioethics Committee of the M.V. Lomonosov Moscow State University Biological department. Animals were sacrificed by quick decapitation (preceded by CO_2_ anesthesia) before electrophysiological experiments.

To obtain neuromuscular preparations containing newly formed NMJs, surgical denervation of *m. EDL* was performed. Under isoflurane anesthesia (5%) peroneal nerve was aseptically crushed approximately 10 mm from its entrance into the muscle. The wound was closed and animals were allowed to recover for 11 days. Such procedure is known to induce muscle reinnervation and synaptic formation, with functional reinnervation starting at days 8–9 after nerve crush (McArdle and Albuquerque, 1973). Synaptic transmission at the newly formed junctions is characterized by lower frequency and longer time course of spontaneous events (miniature endplate potentials, MEPPs) than in functionally mature synapses. Amplitude distribution of MEPPs is also altered and does not fit to normal curve due to the appearance of both low-amplitude events and so-called “giant miniature” events (McArdle and Albuquerque, 1973). Evoked endplate potentials (EPPs) are also altered and demonstrated lower amplitude and quantal content, prolonged time course, and longer synaptic delay compared to mature NMJs (Argentieri et al., 1992; Gaydukov et al., 2019). During short repetitive nerve stimulation (50 Hz, 1 s) no depression of EPPs amplitude or quantal content is observed, but instead a facilitation that is sustained at a higher level than a first EPP in train (Balezina et al., 2007; Bogatcheva and Balezina, 2013).

### Electrophysiology

To study spontaneous synaptic activity, intact neuromuscular preparations were used. To study synaptic activity evoked by nerve stimulation, muscle fibers of neuromuscular preparations were cut to prevent contractions. Immediately after the transverse cutting of muscle fibers, the preparation was thoroughly washed in a large volume (150–200 ml) of Liley solution (Gaydukov et al., 2019) for 60–90 min to prevent the nerve action potential conduction block. As a result, the recorded value of resting membrane potential (RMP) in cut fiber preparations was lower than in intact ones. Such a decrease of RMP prevented the muscle fiber action potential generation and hence muscle contractions in response to nerve stimulation. This procedure allowed to record spontaneous and evoked endplate potentials simultaneously from the same NMJ (Gaydukov et al., 2019).

Isolated neuromuscular preparations of m. EDL or hemidiaphragm were rapidly excised, pinned, and stretched on a Sylgard-coated experimental chamber perfused at 0.5 ml/min by oxygenated (95% O_2_, 5% CO_2_) Liley solution (pH 7.2–7.4). All the experiments were performed at room temperature (20–22°C). The synaptic activity of NMJs was recorded using conventional microelectrode intracellular technique. Glass microelectrodes were filled with 2.5 M KCl (tip resistance 10–20 MΩ) and connected to Axoclamp-2B amplifier (Molecular Devices, San Jose, CA, United States) or Neuroprobe Amplifier Model 1600 (A-M Systems, Sequim, WA, United States). Microelectrodes were inserted into muscle fibers close to the endplate region as judged by the presence of MEPPs with rise times (10–90%) lower than 1 ms when recorded from mature NMJs or 2 ms – from newly formed ones. In addition, RMP was recorded for the further correction of driving force and non-linear summation of EPPs. The signals were digitized using an analog-digital converter E-154 (L-Card, Moscow, Russia) with a PowerGraph 3.3.9 interface and further analyzed using MiniAnalysis software (Synaptosoft, Decatur, GA, United States).

To study the only spontaneous activity of NMJs, MEPPs were recorded from intact neuromuscular preparations with uncut fibers for 120–150 s in each NMJ. To study evoked synaptic activity, the peroneal nerve or phrenic nerve of cut-muscle preparation was stimulated via silver bipolar electrodes by short (1 s) high-frequency (50 Hz) trains of suprathreshold pulses (pulse duration 0.08 ms). To avoid the fatigue of motor synapses and consequent amplitude rundown and changes of the EPP pattern, minimal intervals between stimulus trains were 4–5 min. In each NMJ, MEPPs were recorded for 100 s prior to the stimulation of nerve. The mean value of the MEPP amplitudes recorded within this period was used for the calculation of EPPs quantal content. MEPPs and EPPs from at least five different NMJs were recorded in control from each neuromuscular preparation. After control recordings, the studied drug/s were added to the perfusion solution in a specific order and the activity of different NMJs was recorded within 40–60 min of drugs application. In each experimental series, a minimum of three neuromuscular preparations were used.

### Drugs

We used recombinant human proBDNF (its cleavable form) and BDNF prodomain (purchased from Alomone Labs, Jerusalem, Israel); tertiapin-Q as a selective blocker of inward-rectifier K^+^ channels, iberiotoxin as a selective blocker of the big conductance Ca^2+^-activated K^+^ channels, nitrendipine as a L-type calcium channel blocker, Y-27632 dihydrochloride as a selective inhibitor of ROCK, and (±)-Vesamicol hydrochloride as a direct inhibitor of vesicular ACh transport (all purchased from Tocris, Bio-Techne, Minneapolis, MN, United States); TAT-Pep5 as a p75 receptor signaling inhibitor (purchased from Sigma-Aldrich, United States). BDNF maturation by-products, tertiapin-Q, iberiotoxin, and Y-27632 dihydrochloride were dissolved in deionized water. Stock solutions of all the other drugs were prepared in DMSO (Helicon, Moscow, Russia). The final concentrations of DMSO in the working solution did not exceed 0.01% (v/v). At this concentration, this solvent did not affect the parameters of spontaneous and evoked activity in mouse NMJs.

### Data Analysis and Statistics

Miniature endplate potentials and endplate potentials were detected in digitized intracellular recordings manually using MiniAnalysis software (Synaptosoft, Decatur, GA, United States), no template was used during the analysis. Amplitude and time course of MEPPs and EPPs and the frequency of MEPPs were estimated. The amplitudes of MEPPs from intact neuromuscular preparations with uncut fibers were normalized to −70 mV to correct for the changes in the driving force caused by the voltage shift upon the RMP changes. In cut-muscle preparations, the amplitudes of MEPPs and EPPs were normalized to the membrane potential of −50 mV (Gaydukov et al., 2016). Estimates of the mean quantal content of EPPs were obtained from the ratios of the mean normalized amplitude of EPPs corrected for non-linear summation to the mean normalized amplitude of MEPPs. For correction of EPP amplitudes, we used the formula *A*_corr_ = *A*/[1 − (0.8 V/E)], where *A*_corr_ is the corrected EPP amplitude, *A* – the observed amplitude of EPP, *E* is the difference between the registered RMP and the reversal potential (we considered it 0), and 0.8 is a factor depending on cable properties of muscle fibers (McLachlan and Martin, 1981).

Statistical analysis was performed using GraphPad Prism 7.0 software (GraphPad Software, San Diego, CA, United States). The normality of the parameter distribution was verified using D’Agostino–Pearson test. Based on the test results, the significance between groups was estimated by unpaired Student’s *t*-test when the distributions were normal or by Mann–Whitney test when the distribution was not normal. The Kolmogorov–Smirnov test was used when cumulative probabilities of MEPP amplitudes were compared. Two-way ANOVA (with the *post hoc* Bonferroni correction) was used for the analysis of EPP amplitude and quantal content in trains. Values of *p* < 0.05 were regarded as statistically significant. With the exception of representative original recordings, all data in text and figures are presented as the mean ± standard error of the mean, *n* corresponds to the number of synapses in a particular group.

## Results

### ProBDNF in Reinnervated Motor Synapses

The activity of newly formed NMJs of reinnervated m. EDL was studied in presence of proBDNF (1 nM). During the early reinnervation period, functionally immature synaptic contacts display a reduced frequency of spontaneous quantal ACh release and a slower time course of MEPPs compared to those of mature NMJs (McArdle and Albuquerque, 1973; Gaydukov et al., 2019).

**FIGURE 1:**
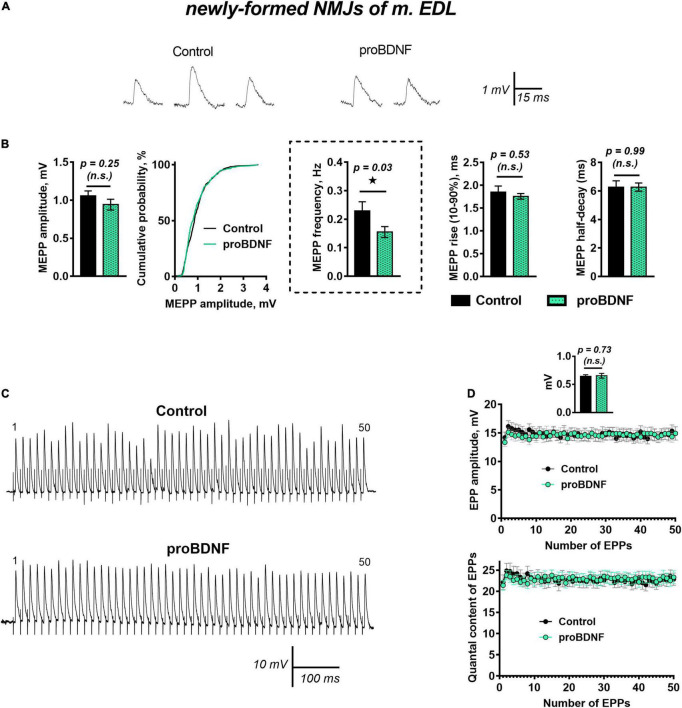
proBDNF in low concentration (1 nM) decreases the rate of spontaneous ACh release at newly formed NMJs but has no effect on evoked ACh release at newly formed NMJs. **(A)** Representative recordings of MEPPs in control (left) and upon application of proBDNF (right). **(B)** Mean MEPP amplitude, cumulative probability plots, frequency and time-course parameters (left to right) in control (*n* = 27) and upon application of proBDNF (*n* = 30). **(C)** Representative recordings of EPPs during a short (1 s) high-frequency (50 Hz) train in control (above) and upon application of proBDNF (below). **(D)** Changes in the EPP amplitude (above) and in the quantal content of EPPs (below) in control (*n* = 19) and in the presence of proBDNF (*n* = 24). Inset shows MEPP amplitudes.

ProBDNF (1 nM) caused a small but statistically significant increase in RMP from −62.19 ± 1.09 mV in control (*n* = 27) to −65.50 ± 1.09 mV (*n* = 30, *p* = 0.04). The amplitude and time course of MEPPs remained unchanged. However, proBDNF decreased the already low frequency of MEPPs in newly formed motor synapses (Figures 1A,B). Evoked synaptic activity was not altered by proBDNF (1 nM): there was no effect on the amplitude and quantal content of EPPs evoked by a short train of stimuli (50 Hz, 1 s) in newly formed synapses (Figures 1C,D).

**FIGURE 2:**
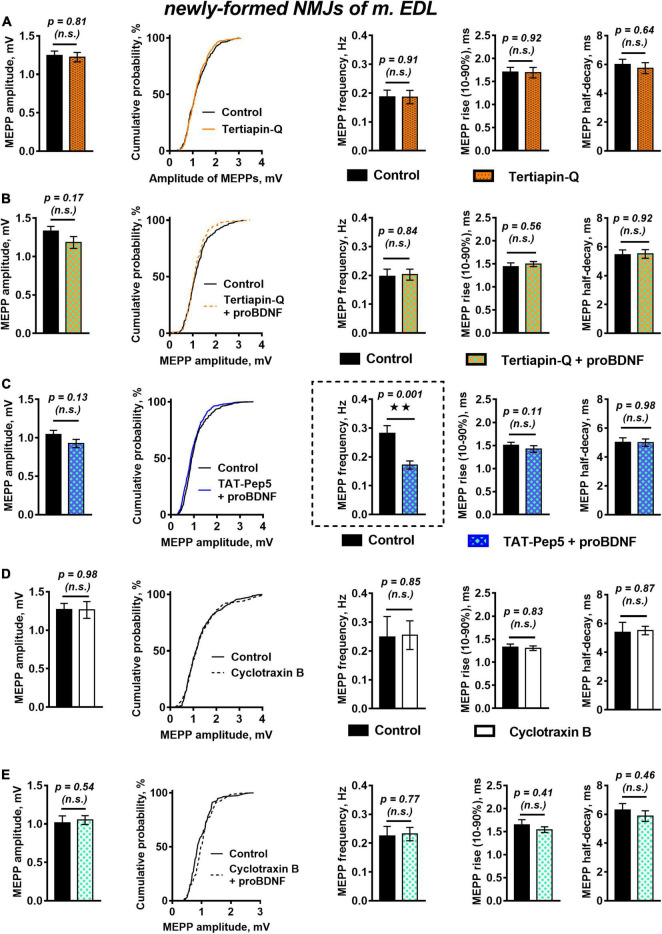
Inhibition of GIRK channels and TrkB receptors but not Rho-GDI-associated signaling of p75 receptors, abolish proBDNF-induced decrease in MEPP frequency newly formed NMJs. **(A)** Mean MEPP amplitude, cumulative probability plots, frequency, and time-course parameters (left to right) in control (*n* = 21) and upon application of GIRK blocker tertiapin-Q (100 nM, *n* = 23). **(B)** Mean MEPP amplitude, cumulative probability plots, frequency, and time-course parameters (left to right) in control (*n* = 17) and upon application of proBDNF (1 nM) in the presence of tertiapin-Q (100 nM, *n* = 26). **(C)** Mean MEPP amplitude, cumulative probability plots, frequency, and time-course parameters (left to right) in control (*n* = 23) and upon application of proBDNF (1 nM) in the presence of Rho-GDI-associated p75 signaling inhibitor TAT-Pep5 (1 μM, *n* = 31). **(D)** Mean MEPP amplitude, cumulative probability plots, frequency, and time-course parameters (left to right) in control (*n* = 15) and upon application of TrkB antagonist cyclotraxin B (100 nM, *n* = 19). **(E)** Mean MEPP amplitude, cumulative probability plots, frequency, and time-course parameters (left to right) in control (*n* = 19) and upon application of proBDNF (1 nM) in the presence of cyclotraxin B (100 nM, *n* = 28).

A likely reason for the observed downregulation of MEPPs frequency in the presence of proBDNF could be hyperpolarization of the cell membrane (Lupica et al., 2001). Hyperpolarization of sarcolemma or presynaptic membrane is often caused by an increase in outward potassium currents. Among numerous others, these could be currents through G-protein-coupled inwardly rectifying potassium channels (GIRK), which stabilize the cell RMP. Along with activation by G-protein-coupled receptors (Hibino et al., 2010), modulation of GIRK by neurotrophins has also been described (Rogalski et al., 2000; Coulson et al., 2008). To test the possible involvement of GIRK in proBDNF-induced negative regulation of spontaneous activity in reinnervated NMJs, we used the selective GIRK blocker tertiapin-Q (100 nM). Tertiapin-Q did not change the RMP of muscle fibers, the frequency, amplitude, or time course of MEPPs (Figure 2A). However, in the presence of tertiapin-Q proBDNF had lost the ability to shift the RMP of muscle fibers toward hyperpolarization (RMP was −60.53 ± 1.38 mV in control (*n* = 17) and −61.12 ± 1.19 mV (*n* = 26, *p* = 0.40) under proBDNF with tertiapin-Q) and to reduce MEPPs frequency (Figure 2B). This suggests that GIRK may modulate RMP of post- and pre-synaptic membranes and inhibit spontaneous quantal ACh release, and thereby may mediate inhibitory effects of proBDNF in reinnervated NMJs of m. EDL.

The involvement of GIRK in the suppression of MEPPs frequency and muscle fibers hyperpolarization implies activation of certain receptors by proBDNF and subsequent triggering of signaling pathways that result in activation of GIRK. The most likely candidate for proBDNF-activated receptor is p75, which belongs to the tumor growth factor receptor family (Malik et al., 2021). p75 expression was shown at the pre- and post-synaptic levels both in developing and mature neuromuscular junctions (Garcia et al., 2010a,b, 2011).

We then used a peptide inhibitor of p75 signaling associated with Rho GDP-dissociation inhibitor (Rho-GDI), TAT-Pep5 (1 μM), to reveal the possible involvement of p75 activation as an initial step in the inhibitory effect of proBDNF on MEPPs frequency in newly formed NMJs. Surprisingly, we found that TAT-Pep5 did not prevent the action of proBDNF (1 nM): in its presence proneurotrophin had a hyperpolarizing effect on the RMP of muscle fibers [it was −66.74 ± 0.90 mV (*n* = 23) and −69.42 ± 0.74 mV (*n* = 31, *p* = 0.03) in control and under proBDNF with TAT-Pep5, respectively] and significantly reduced the frequency of MEPPs (Figure 2C).

Despite it is commonly recognized that proneurotrophins act predominantly on p75, we still decided to clarify the possible involvement of TrkB in proBDNF effects in newly formed NMJs. We used cyclic peptide antagonist of TrkB – cyclotraxin B (100 nM). Cyclotraxin B did not influence spontaneous MEPPs parameters in regenerating NMJs (Figure 2D). Surprisingly, it prevented the ability of proBDNF to reduce the frequency of MEPPs and to increase RMP (Figure 2E). This data, together with the inability of TAT-Pep5 to abolish the negative effects of proBDNF, may indicate possible non-canonical action of proBDNF on TrkB receptors in newly formed synapses.

Since the cleavable form of proBDNF was used in our experiments, we cannot rule out the possibility that the observed effects could be partly due to the products of its hydrolysis, including the BDNF prodomain. In this regard, we further investigated the role of the BDNF prodomain in the regulation of ACh release in regenerating motor synapses.

### Brain-Derived Neurotrophic Factor Prodomain in Reinnervated Neuromuscular Junctions

**FIGURE 3:**
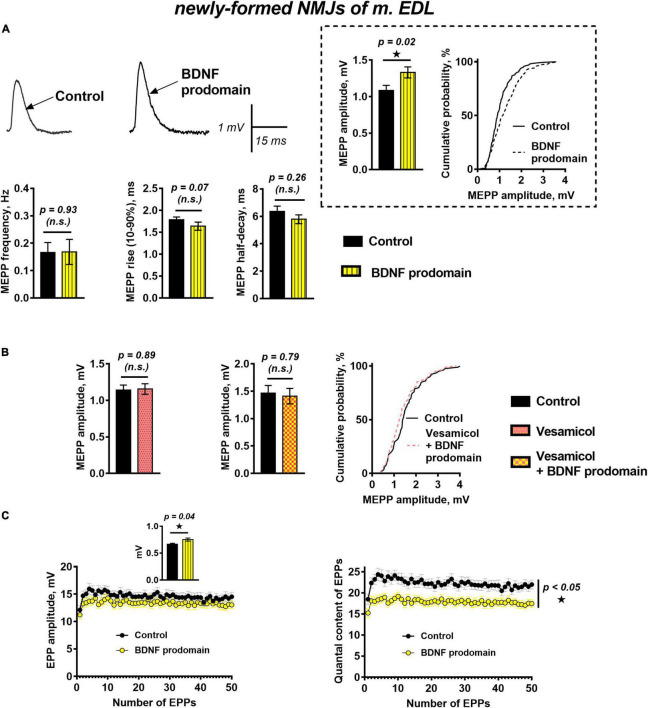
BDNF prodomain in low concentration (1 nM) pre-synaptically increases ACh quantal size and simultaneously induces oppositely directed presynaptic effects affecting the evoked ACh release at newly formed NMJs of reinnervated mouse m. EDL. **(A)** Representative recordings of MEPPs (left above) and mean MEPP amplitude and cumulative probability plots (right above), frequency and time-course parameters (left to right below) in control (*n* = 20) and upon application of BDNF prodomain (*n* = 22). **(B)** Mean MEPP amplitude in control (*n* = 16) and during inhibition of vesicular ACh transporter by vesamicol (1 μM, *n* = 17) (left) and mean MEPP amplitude and cumulative probability plots in control (*n* = 15) and upon application of BDNF prodomain in the presence of vesamicol (*n* = 16). **(C)** Changes in the EPP amplitude (left) and their quantal content (right) in control (*n* = 31) and in the presence of BDNF prodomain (*n* = 41). Inset shows MEPP amplitudes.

The effects of the BDNF prodomain (1 nM) on neurotransmission in newly formed synapses of m. EDL differed drastically from the effects of proneurotrophin. The RMP of muscle fibers was −64.85 ± 0.84 mV (*n* = 20) in control and −63.27 ± 0.85 mV (*n* = 22, *p* = 0.19) under BDNF prodomain. The frequency and the time course of MEPPs in the presence of the BDNF prodomain also matched the control values. At the same time, the unexpected effect – a statistically significant increase in the amplitude of MEPPs by 30% – was observed (Figure 3A).

The potentiation of the MEPP amplitudes by the BDNF prodomain could be due to an increase in the amount of ACh molecules stored in each synaptic vesicle. To test this hypothesis, we used vesamicol (1 μM), an inhibitor of the vesicular ACh transporter. Vesamicol is known to have no effect on the amplitude of uniquantal MEPPs in mature NMJs (Gaydukov et al., 2016). It did not change the amplitude of MEPPs in newly formed motor synapses either. However, vesamicol completely abolished the ability of the BDNF prodomain to increase the amplitude of MEPPs in reinnervating NMJs (Figure 3B). The obtained data suggest that the BDNF prodomain may activate presynaptic receptors, enhance ACh loading into synaptic vesicles and, thereby, increase ACh quantal size. Interestingly, this effect of prodomain is very similar to the effect of mature BDNF on the amplitude of MEPPs in newly formed NMJs that we recently discovered (Gaydukov et al., 2019).

A study of evoked ACh release during short rhythmic trains of EPPs (50 Hz, 1 s) in newly formed synapses showed that BDNF prodomain significantly reduced the quantal content of EPPs throughout the train by 20%, but the amplitude of EPPs was not changed. At the same time, prodomain increased the amplitude of MEPPs recorded before nerve stimulation from the same synapses where EPPs were recorded (Figure 3C). It seems like the BDNF prodomain induces the simultaneous development of two oppositely directed presynaptic effects (an increase of ACh quantal size and decrease of the quantal content of multiquantal EPPs) which allows to maintain the already low amplitude of EPPs in reinnervated synapses on a steady control level.

Thus, we found that in motor synapses undergoing structural and functional remodeling during muscle reinnervation, both proneurotrophin and BDNF prodomain have acute effects on various parameters of ACh quantal release, which do not match the effects of mature BDNF.

Of course, the question remained whether proBDNF and BDNF prodomain would influence synaptic transmission in functionally mature mouse neuromuscular junctions in the same way.

### ProBDNF and Brain-Derived Neurotrophic Factor Prodomain in Mature Neuromuscular Junctions

**FIGURE 4:**
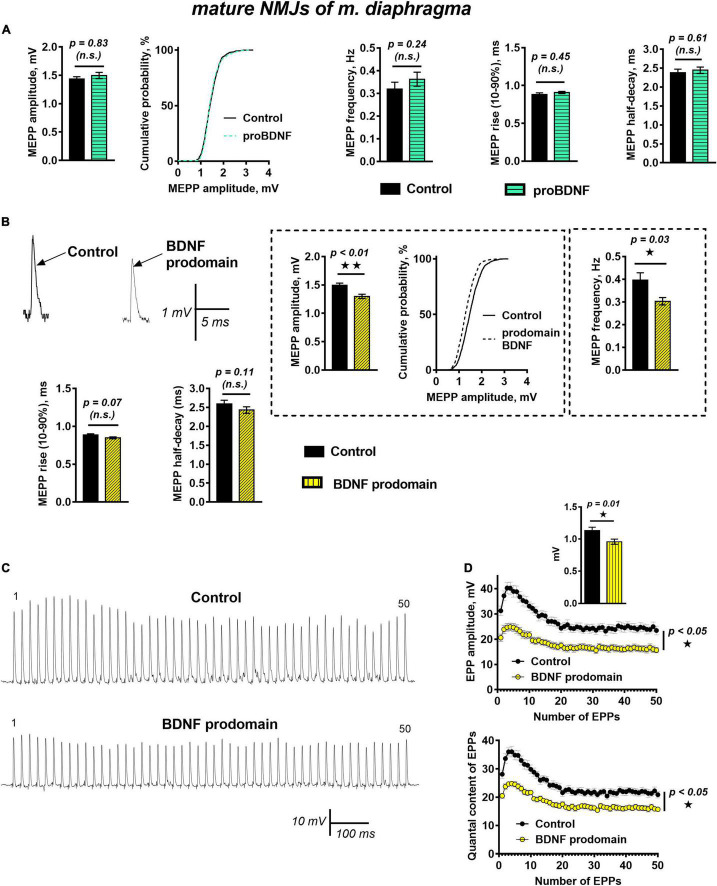
BDNF prodomain (1 nM) but not proBDNF (1 nM), induces strong inhibition of spontaneous end evoked ACh release at mature NMJs. **(A)** Mean MEPP amplitude, cumulative probability plots, frequency, and time-course parameters (left to right) in control (*n* = 19) and upon application of proBDNF (*n* = 26). **(B)** Representative recordings of MEPPs (top left) and mean MEPP amplitude, cumulative probability plots, frequency, (top right) and their time-course parameters (bottom) in control (*n* = 23) and upon application of BDNF prodomain (*n* = 33). **(C)** Representative recordings of EPPs during a short (1 s) high-frequency (50 Hz) train in control (above) and upon application of BDNF prodomain (below). **(D)** Changes in the EPP amplitude (above) and in the quantal content of EPPs (below) in control (*n* = 22) and in the presence of proBDNF (*n* = 21). Inset shows MEPP amplitudes.

Unexpectedly, unlike in reinnervated synapses of m. EDL, proBDNF (1 nM) did not cause significant changes of muscle fibers RMP, frequency, amplitude, or time course of MEPPs in functionally mature motor synapses of mouse diaphragm when exposed for 40–60 min (Figure 4A). This contrast may be associated with changes in proneurotrophin activity in mature motor synapses. These include a decrease in proBDNF expression (compared with the post-denervation stage), changes in the expression of receptors, and intra- and extra-cellular enzymes required for proBDNF cleavage, both in muscle fibers and in motor nerve terminals (Meeker and Williams, 2014; Aby et al., 2021).

Even more unexpected was the fact that in mature NMJs BDNF prodomain (1 nM) induced numerous changes in synaptic transmission resulting in inhibition of spontaneous and evoked quantal ACh release. BDNF prodomain caused an acute decrease in the frequency of MEPPs by 28%, along with a small but statistically significant decrease in the amplitude of MEPPs by 16%. The time course of MEPPs and RMP of diaphragm muscle fibers remained unaltered (Figure 4B).

During short trains of EPPs (50 Hz, 1 s) BDNF prodomain did not influence the basic pattern of EPP train (i.e., initial facilitation, followed by depression and steady-state level of ACh release – plateau), but led to a pronounced decrease in the amplitude of EPPs throughout the train. This was quite predictable, given that the amplitude of uniquantal MEPPs recorded from the same synapses as EPPs before nerve stimulation was also decreased. But along with that, the quantal content of EPPs in train was lowered by 25% in the presence of BDNF prodomain (Figures 4C,D).

Hence, we received novel evidence of the strong inhibitory action of the BDNF prodomain on synaptic transmission in functionally mature NMJs. Seemingly, this inhibition may occur both on presynaptic (by decreasing MEPPs frequency and EPPs quantal content) and maybe post-synaptic (by lowering the amplitude of MEPPs and EPPs) levels.

Next, it was necessary to reveal which targets and signaling pathways mediate the negative effect of the BDNF prodomain on synaptic transmission in mature NMJs.

The decrease of EPPs quantal content by prodomain could be due to an enhanced activity of presynaptic potassium channels involved in the regulation of evoked ACh release. One of the likely candidates could be calcium-activated potassium channels of BK-subtype, which may serve as a potential target for neurotrophin-mediated regulation (Cao et al., 2012).

To test this assumption, we blocked BK channels with iberiotoxin (100 nM). Along with that, L-type voltage-gated calcium channels were blocked with nitrendipine (1 μM). It was necessary because otherwise blocking BK channels would have led to an enhancement of L-type calcium channels activity and to the increase of EPPs amplitude and quantal content (Flink et al., 2003; Gaydukov et al., 2009), thus masking the inhibitory influence of BDNF prodomain.

**FIGURE 5:**
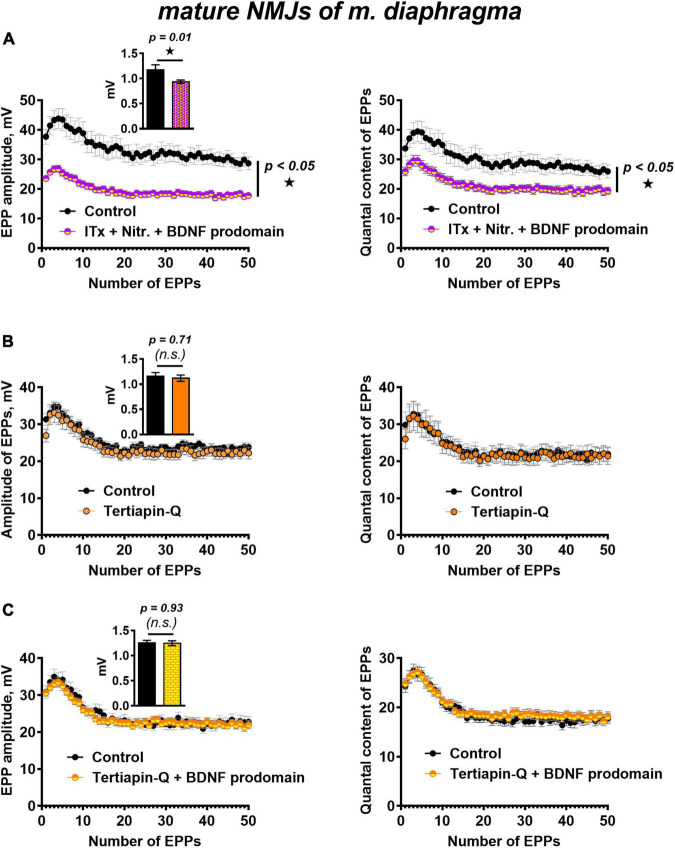
BK channels do not but GIRK channels mediate BDNF prodomain-induced inhibition of evoked ACh release at mature NMJs. **(A)** Changes in the EPP amplitude (left) and in the quantal content of EPPs (right) in control (*n* = 15) and upon BDNF prodomain (1 nM) in the presence of BK-blocker iberiotoxin (ITx, 100 nM) with L-type Ca^2+^-channel blocker nitrendipine (Nitr., 1 μM) (*n* = 21). **(B)** Changes in the EPP amplitude (left) and in the quantal content of EPPs (right) in control (*n* = 15) and in the presence of GIRK blocker tertiapin-Q (100 nM, *n* = 17). **(C)** Changes in the EPP amplitude (left) and in the quantal content of EPPs (right) in control (*n* = 16) and upon BDNF prodomain (1 nM) in the presence of tertiapin-Q (*n* = 19). Insets show MEPP amplitudes.

We have previously shown that simultaneous blocking of BK potassium channels and L-type calcium channels maintains evoked ACh release at a control level throughout the EPP train (Gaydukov et al., 2009). In this study, the presence of iberiotoxin and nitrendipine BDNF prodomain (1 nM) still caused a decrease in the amplitude and the quantal content of EPPs in the short train. Hence, the BDNF prodomain retained its inhibitory effect on synaptic transmission when potassium current through BK channels was blocked (Figure 5A). This allowed to rule out BK channels as a possible target of BDNF prodomain activity in mature motor synapses.

In this regard, we further investigated whether GIRK channels can mediate the inhibitory effects of the BDNF prodomain. GIRK channel blocker tertiapin-Q (100 nM) had no effect on the parameters of evoked ACh release (Figure 5B), but it completely prevented the decrease in the amplitude and quantal content of EPPs caused by BDNF prodomain (1 nM) in mature NMJs (Figure 5C). Apparently, GIRK channels, known as modulators of presynaptic RMP and action potential of nerve terminals in CNS (Meneses et al., 2015; Vázquez-Vázquez et al., 2020), may be downstream targets of BDNF prodomain and mediate its inhibitory effects on evoked ACh release in mouse motor synapses.

**FIGURE 6:**
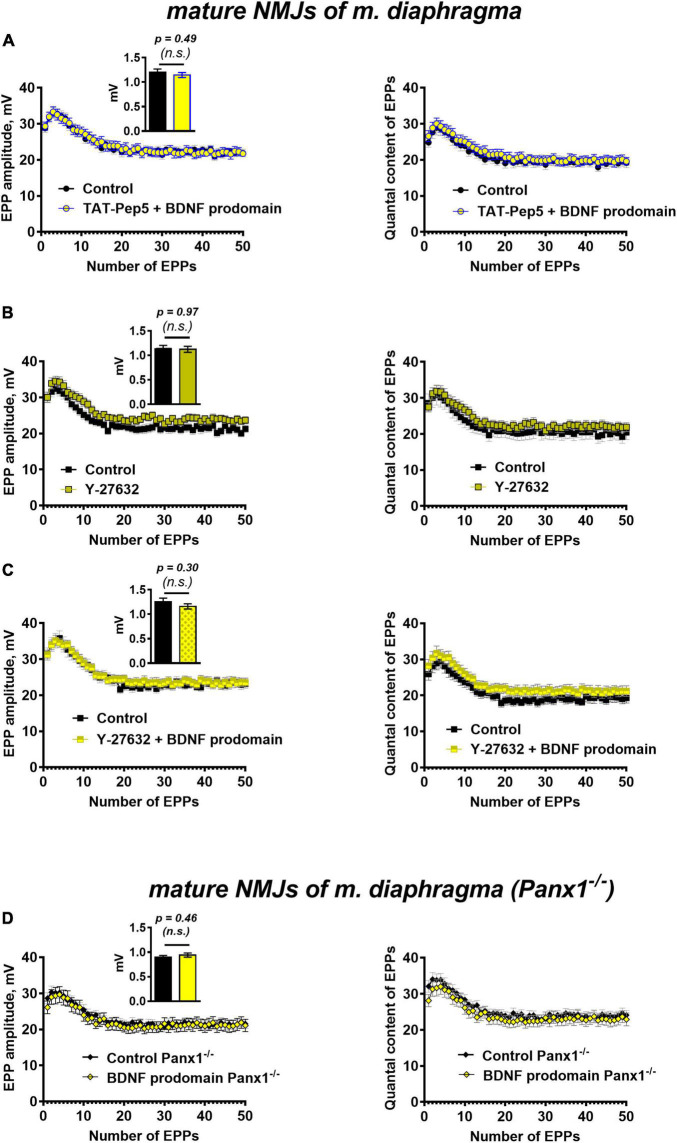
p75 receptors and Rho-signaling pathway underlie BDNF prodomain-triggered inhibition of evoked ACh release at mature NMJs. Moreover, the inhibitory effect of the BDNF prodomain (1 nM) on the evoked neuromuscular transmission depends on the endogenous activity of synaptic purinoreceptors at mature NMJs. **(A)** Changes in the EPP amplitude (left) and in the quantal content of EPPs (right) in control (*n* = 20) and upon BDNF prodomain (1 nM) in the presence of Rho-GDI-associated p75 signaling inhibitor TAT-Pep5 (1 μM, *n* = 21). **(B)** Changes in the EPP amplitude (left) and in the quantal content of EPPs (right) in control (*n* = 17) and in the presence of ROCK inhibitor Y-27632 (3 μM, *n* = 21). **(C)** Changes in the EPP amplitude (left) and in the quantal content of EPPs (right) in control (*n* = 15) and upon BDNF prodomain (1 nM) in the presence of Y-27632 (*n* = 21). **(D)** Changes in the EPP amplitude (left) and in the quantal content of EPPs (right), registered from NMJs of Panx1^–/–^ mice in control (*n* = 24) and in the presence of BDNF prodomain (*n* = 22). Insets show MEPP amplitudes.

Currently, few literature data link the specific regulatory activity of BDNF prodomain in CNS with the activation of p75 receptors (Mizui et al., 2016; De Vincenti et al., 2019; Kojima et al., 2019). In this study, we have shown that in motor synapses inhibition of Rho-GDI-associated p75 signaling with TAT-Pep5 peptide (1 μM) completely abolished inhibitory effects of BDNF prodomain on evoked ACh release. The amplitude and the quantal content of EPPs in trains under these conditions did not differ from the control level (Figure 6A).

Since the action of the BDNF prodomain was found to be sensitive to the inhibition of p75 signaling, associated with Rho-GDI, we further tested the possible involvement of the Rho-signaling pathway in the effects of the BDNF prodomain. The Rho-associated protein kinase (ROCK) inhibitor, Y-27632 (3 μM), that was used for the first time in the analysis of ACh release in motor synapses, judging by the lack of literature data. The inhibition of ROCK did not cause any significant changes in the parameters of EPPs in mature diaphragm synapses (Figure 6B). But similarly to the action of tertiapin-Q and TAT-Pep5, Y-27632 also completely abolished the prodomain-induced decrease in the amplitude and quantal content of EPPs in short trains (Figure 6C).

Taken together, these data suggest that in mature motor synapses activation of p75 by BDNF prodomain triggers the Rho-signaling pathway and the likely target of this cascade are GIRK channels.

However, activation of GIRK in CNS synapses is usually triggered by metabotropic G-protein-coupled receptors (GPCRs) (Fernández-Fernández and Lamas, 2021). At the same time, neurotrophin receptors are capable of transactivation by GPCRs (Kilpatrick and Hill, 2021) and functional interaction with purinoreceptors (Pousinha et al., 2006; Tomàs et al., 2017; Gaydukov et al., 2019). In this regard, we further investigated whether the BDNF prodomain may activate GIRK channels and inhibit ACh release on its own, or does this require the involvement of certain GPCRs? Presynaptic A1 receptors for adenosine and P_2_Y_13_ receptors for ATP are known to inhibit synaptic transmission at mammalian NMJs (Perissinotti and Uchitel, 2010; Guarracino et al., 2016; Miteva et al., 2017) and may take on the role of such GPCRs.

To test the possible involvement of these purinoreceptors in the inhibitory activity of the BDNF prodomain in motor synapses, we applied a procedure for their deactivation. To do this, instead of traditional pharmacological inhibition with receptor antagonists, we used neuromuscular preparations obtained from pannexin 1 knock-out mice (Panx1^–/–^). Pannexin 1 hemichannels are capable of releasing intracellular ATP into the synaptic cleft. We have previously shown the obligatory role of endogenous ATP, released into the synaptic cleft via pannexins, in the activation of inhibitory presynaptic A1- and P_2_Y_13_ purinoreceptors (along with vesicular ATP released with synaptic vesicles) in motor synapses (Miteva et al., 2017, 2018, 2020). In Panx1^–/–^ mice the “pannexin source” of ATP is abolished. Synaptic transmission at NMJs of these mice remains unaltered in comparison with wild-type motor synapses, but presynaptic A1 and P_2_Y_13_ receptors are insufficiently activated by endogenous purines, and their inhibitory effect on ACh release is eliminated (Miteva et al., 2017). Hence, in the last series of experiments we studied the effects of the BDNF prodomain in Panx1^–/–^ mice. BDNF prodomain failed to a decrease in the amplitude and quantal content of EPPs in short trains (Figure 6D). This may indicate that BDNF prodomain-induced inhibition of ACh release requires the simultaneous activation of certain metabotropic purinoreceptors in NMJs. They may enable the downstream activation of GIRK channels after p75-mediated action of the BDNF prodomain. But in contrast with CNS synapses, where the involvement of A1 adenosine receptors in the activation of GIRK and inhibition of neurotransmitter release has already been shown (Kim and Johnston, 2015; Hill et al., 2020), the types of metabotropic purinoreceptors involved in modulation of GIRK activity in motor synapses remain an open question.

## Discussion

In this work, we report novel data discovering the previously unknown activity of two peptide byproducts of BDNF processing (proBDNF and BDNF prodomain) in mouse neuromuscular junctions. These signaling molecules are involved in the regulation of ACh quantal release and their effects in most cases are different from those of mature BDNF. In particular, they suppress the quantal ACh release of ACh. The activity of mature neurotrophin has recently gained more attention due to its role as a myokine (Phillips et al., 2014), and a retrograde modulator of ACh release with a potentiating effect on spontaneous and evoked synaptic transmission (Hurtado et al., 2017; Gaydukov et al., 2019). BDNF activates presynaptic TrkB receptors and triggers a number of signaling pathways in motor nerve terminals, leading to stimulation of vesicular ACh transport and to an increase in quantal size, frequency, and amplitude of MEPPs, and quantal content of EPPs. Potentiating effects of BDNF were also revealed in functionally immature synapses, formed during muscle fibers reinnervation (Gaydukov et al., 2019).

In newly formed synapses of m. EDL proBDNF at low nanomolar concentration caused a decrease in the MEPPs frequency and an increase in the RMP of muscle fibers. This inhibitory effect of proBDNF on spontaneous ACh release is different from the potentiating effect of mature BDNF on the amplitude of post-synaptic potentials in regenerating motor synapses. This partly coincides with the antagonistic effects of these signaling molecules in neonatal motor synapses, where an inhibitory effect of proBDNF on spontaneous and evoked ACh release due to activation of presynaptic p75 receptors by proneurotrophin was observed (Lu, 2003; Yang et al., 2009; Je et al., 2012, 2013).

However, in the newly formed synapses, we failed to prevent the inhibitory effect of proBDNF on the frequency of MEPP using TAT-Pep5, an inhibitor of p75 signaling associated with Rho-GDI. Perhaps in this case, given the targeted influence of proBDNF only on the frequency of spontaneous secretion, its effects were associated with the activation of some other component of the neurotrophin signaling. Here, we assume unexpected modulation of spontaneous ACh release by proneurotrophin and TrkB signaling. Notably, the effect of TrkB activation by proBDNF is very different from the one induced by mature BDNF in newly formed NMJs (Gaydukov et al., 2019). Another new and unexpected fact is the relation of the TrkB-mediated proBDNF-induced inhibitory effect on the frequency of MEPPs and an increase in muscle fiber RMP with the activity of GIRK channels in newly formed NMJs. Examples are known in CNS, when the activation of GIRK in nerve terminals leads to downregulation of neurotransmitter release (Meneses et al., 2015; Vázquez-Vázquez et al., 2020). However, the specific signaling mechanism of proBDNF-induced inhibition of spontaneous ACh release involving GIRK activation in newly formed NMJs remains to be discovered in further experiments.

Along with proBDNF, the activity of BDNF prodomain in newly formed synapses was studied for the first time. It turned out that the BDNF prodomain has multidirectional effects on the ACh quantal release: it acts synergistically with BDNF and stimulates an increase of ACh quantal size, but it reduces the quantal content of EPPs. The partial similarity of prodomain and mature BDNF effects may be due to the ability of p75 activated by prodomain to act as co-receptors and modulators of TrkB activity (Huang and Reichardt, 2003; Haddad et al., 2017; Malik et al., 2021).

As we have shown earlier, activation of TrkB receptors by mature BDNF triggers a complex signaling pathway involving mitogen-activated protein kinases, leading to an increase of the ACh quantal size (Gaydukov et al., 2018, 2019). The decrease of EPPs quantal content in the presence of prodomain corresponds to modern data about the inhibitory effects of proBDNF (and BDNF prodomain) on neurotransmitter release and the development of forms of synaptic plasticity that downregulate it in CNS synapses and regenerating neuromuscular junctions (Je et al., 2012, 2013; Mizui et al., 2016; De Vincenti et al., 2019; Kojima et al., 2019).

It cannot be ruled out that the multidirectional action of proBDNF and BDNF prodomain is the result of independent regulation of different parameters of spontaneous and evoked ACh release. This may be due to the formation of a set of signaling molecules (proBDNF and its cleavage products) in the synaptic cleft that activate different types of neurotrophin receptors in newly formed synapses. Anyway, the final identification of the signaling mechanisms that mediate complex and multidirectional effects of the BDNF prodomain on ACh release in reinnervated NMJs requires further analysis.

Along with regenerating synapses, we studied mature mouse diaphragm NMJs and also revealed their reactivity to BDNF processing products, at least to the BDNF prodomain.

We found that proBDNF action in mature motor synapses does not influence the parameters of spontaneous ACh release. During the development and maturation of motor neurons and muscles, a decrease in endogenous proBDNF is observed both in motor neurons and in mature muscle fibers (Yang et al., 2009; Je et al., 2012). This suggested that expression, release into the synaptic cleft, and activity of proneurotrophin become unnecessary in mature NMJs. Accordingly, one could think that in the absence of regulatory activity of proBDNF (at least in relation to spontaneous ACh release), the product of its hydrolysis, BDNF prodomain, would also be ineffective.

However, unexpectedly, we discovered a previously unknown wide range of vivid inhibitory effects of BDNF prodomain on spontaneous uniquantal and evoked synchronous multiquantal ACh release in mature NMJs: a significant decrease in the frequency of MEPPs, a decrease in the quantal content of EPPs in short-term high-frequency trains, and a decrease in the amplitude of post-synaptic potentials in the presence of BDNF prodomain.

Taking into account that the BDNF prodomain is formed *in vivo* as a result of proBDNF cleavage (Wang et al., 2021), the presence of regulatory activity of prodomain, but not of its predecessor, seems, at first glance, paradoxical. This could be the result of rapid cleavage of exogenous proBDNF in the synaptic cleft of mature endplates and the formation of two peptide products – BDNF prodomain and mature BDNF in equimolar concentrations. As a result, each of them, by triggering their own signaling pathways, can mutually neutralize their oppositely directed effects on ACh release, which does not allow revealing specific effects of proBDNF. However, such an explanation for the inefficiency of exogenous proBDNF in mature NMJs, especially given a pronounced activity of both mature BDNF and its prodomain, is still purely hypothetical and requires further careful analysis.

Thus, we have shown for the first time that not only in motor synapses undergoing remodeling and functional maturation but also in fully formed NMJs, proBDNF or its related prodomain are capable of inhibiting spontaneous and evoked ACh release, in the latter case with the involvement of p75 receptors.

Currently, the BDNF prodomain as a separate signal molecule (consisting of about 110 amino acids) has been described in a number of neurons in CNS (Zanin et al., 2017). It has been shown to be stored in large dense core vesicles with BDNF and released along with it into synaptic cleft from the nerve terminals (Dieni et al., 2012). It is considered that the BDNF prodomain can also be produced from proBDNF in the synaptic cleft, where proneurotrophin-cleaving proteases are present, which is also correct for neuromuscular junctions (Vansaun et al., 2003; Je et al., 2012).

A high level of expression and activity of the BDNF prodomain as a factor that induces the growth cone retraction was noted in developing synapses (Anastasia et al., 2013). At the same time, exogenous BDNF prodomain in hippocampal slices rapidly increased long-term depression in a p75-mediated way, i.e., it functioned as a fast-acting signaling molecule (Kojima et al., 2019). But the exact mechanism of this inhibitory effect of BDNF prodomain on synaptic transmission in the hippocampus remains unclear.

In this study, we discovered not only a previously unknown acute regulatory activity of BDNF prodomain, aimed at multitarget inhibition of ACh release in mature NMJs. The results of our experiments shed light on a possible mechanism of this prodomain effect associated with the activation of p75 receptors, triggering of a signaling pathway involving ROCK, and, ultimately, potentiation of GIRK channels.

The discovery of GIRK channels involved in the negative regulation of ACh quantal release in NMJs by two previously unexplored BDNF-related signaling molecules is the priority of this work.

Presynaptic GIRKs have been described in CNS synapses (Ponce et al., 1996; Luján and Aguado, 2015). The involvement of these inward-rectifying potassium channels in the inhibition of spontaneous and evoked synaptic transmission, which is prevented by tertiapin-Q, is thought to result from the activation of various presynaptic GPCRs. Upon activation of such GPCRs, G-protein βγ-subunits can directly activate GIRK (Lüscher and Slesinger, 2010). This, in turn, can lead to hyperpolarization of presynaptic membrane and suppression of Ca^2+^-dependent release of neurotransmitter from nerve terminals (Dascal, 1997; Sun et al., 2001; Yum et al., 2008; Jeremic et al., 2021).

Interestingly, in the motor synapse, despite the presence of a wide variety of potassium channels (Meir et al., 1999; Roncarati et al., 2001; Flink et al., 2003; Brooke et al., 2004; Dodson and Forsythe, 2004; Gaydukov et al., 2009, 2014; Wang et al., 2020), the presence of GIRK and their involvement in the regulation of ACh release in response to the action of various modulators of synaptic transmission has not been described so far.

In neurons, increase in activity of GIRK channels may occur not only upon stimulation of GPCRs but also under the action of neurotrophins. However, in such publications, the activation of p75 receptors by neurotrophins and the development of subsequent processes, including GIRK activation and the leakage of potassium cations from cells, serve another purpose: triggering cell death (which is a slower process compared to the regulation of synaptic transmission) (Coulson et al., 2008) or the development of certain pathological conditions. For example, the p75/GIRK tandem is involved in the development of Alzheimer’s disease (May et al., 2017).

It should be noted that the idea of the role of p75 receptors activated by neurotrophins as death receptors that trigger degeneration and apoptosis (with the involvement of GIRK), has undergone a thorough revision in recent years. There is a growing body of evidence for the polyfunctional activity of p75 as pleiotropic receptors capable of participating in multidirectional neuroplasticity processes (Nykjaer et al., 2005; Meeker and Williams, 2014; Riffault et al., 2014; Malik et al., 2021).

This is also supported by data demonstrating that p75 is a target receptor activated by two BDNF-related molecules, proBDNF and BDNF prodomain, that can be involved in the mechanisms of functional inhibition of synaptic transmission in CNS (Mizui et al., 2016; Kojima et al., 2019) and in juvenile NMJs (Yang et al., 2009; Je et al., 2012, 2013).

Here, we have shown for the first time that in mature NMJs the inhibitory effect of BDNF prodomain on ACh quantal release depends on the activation of presynaptic p75 receptors and GIRK channels. Judging by the loss of prodomain effects in presence of an inhibitor of p75 signaling associated with Rho-GDI (TAT-Pep5) or an inhibitor of ROCK (Y-27632), the Rho-signaling pathway may be involved. The role of ROCK in motor synapses has not yet been described. However, there is evidence about its participation in a number of signaling cascades accompanying p75 activation by neurotrophins (Guiler et al., 2021; Malik et al., 2021).

This may indicate a possible regulatory role of p75 receptors, which is not associated with the triggering of destructive or pathological processes in mature NMJs. We cannot rule out that p75 in functional tandem with GIRK is able to provide a previously unknown mechanism of specific inhibitory autoregulation of synaptic transmission by antero- and/or retrograde action of neurotrophins. This can occur along with or in conjunction with other autoregulators of ACh release in NMJs, such as synaptic adenosine or ATP (Pousinha et al., 2006; Gaydukov et al., 2019).

Currently, it is known that autoregulatory activity of ATP exists in motor synapses. ATP is released along with ACh from synaptic vesicles and exerts its effects along with its hydrolysis product adenosine. Acting on their numerous receptors, both ATP and adenosine are able to bidirectionally regulate synaptic transmission, and normally the activity of purine and adenosine receptors that inhibit the release of ACh dominates (Hamilton and Smith, 1991; Ribeiro et al., 1996; Guarracino et al., 2016). We have recently shown that vesicular ATP alone is not sufficient for such activation: the presence of another pool of endogenous ATP is required. This is ATP, released by perisynaptic cells via pannexins 1, described in muscle, glial cells, and axons. We have shown that ATP outflux via pannexins is strictly required for the efficient activation of presynaptic inhibitory A1-receptors for adenosine and P_2_Y_13_-receptors for ATP. When the activity of pannexins is abolished, the inhibitory activity of the mentioned purinoreceptors in NMJs also disappears (Miteva et al., 2017). Thus, a new method was found to selectively weaken the predominantly inhibitory effect of certain types of purinoreceptors on ACh release in motor synapses. The use of Panx1^–/–^ mice in this study made it possible to reduce the level of endogenous ATP/adenosine in motor synapses to values at which there is no efficient activation of inhibitory presynaptic A1- and P_2_Y_13_-receptors. Consequently, signaling cascades that inhibit the evoked release of ACh are not triggered. Unexpectedly, it turned out that in this case not only the absence of inhibitory effects from purinergic receptors is observed but the ability of the BDNF prodomain to decrease the parameters of ACh release is also lost.

Until now, only cooperativity between mature BDNF and A2A-adenosine receptors have been described in motor synapses and it is aimed toward enhancement of neuromuscular transmission (Pousinha et al., 2006; Assaife-Lopes et al., 2014) including the increase in ACh quantal size in NMJs (Gaydukov et al., 2019). In this work, we have shown for the first time that the inhibitory effect of BDNF-related product, BDNF prodomain also involves functional coupling, but with other purinoreceptors that provide inhibitory regulation of ACh release.

However, identification of the mechanisms underlying such interaction of receptors on the pre- or post-synaptic membrane, of course, requires further research.

## Summary

In this work, we described the effects and made substantial progress in revealing the mechanisms of regulatory action of proBDNF and BDNF prodomain on synaptic transmission in NMJs at the stage of their post-traumatic regeneration or in a functionally mature state. Both products of BDNF processing, studied for the first time in these objects, have a predominantly inhibitory effect on ACh release, in contrast to the potentiating effect of BDNF. It was shown for the first time that in mature motor synapses BDNF prodomain downregulates ACh release by activation of p75 receptors, the Rho-signaling pathway, and GIRK channels in interaction with synaptic purinoreceptors. Such inhibitory activity has so far been revealed only in for exogenous products of BDNF processing. However, along with BDNF, endogenous products of its processing may form in the synaptic cleft or be released there. In this case, it is tempting to suggest that a set of signaling molecules, including endogenous BDNF and by-products of its maturation, like most known regulators (neurotransmitters, neuromodulators, hormones), is capable of homeostatic antero- or retrograde modulation of synaptic transmission in intact NMJs through oppositely directed influences involving a complex of receptors and signaling pathways.

Such ideas, however, are hypothetical and require further research and verification.

## Data Availability Statement

The data that support the findings of this study are available from the corresponding author upon reasonable request.

## Ethics Statement

The animal study was reviewed and approved by the Bioethics Committee of the M.V. Lomonosov Moscow State University Biological Department.

## Author Contributions

AG and OB responsible for conceptualization research design. PB, AIM, EP, ASM, and AG conducted the electrophysiological experiments and analyzed the data. AG prepared the figures and received funding. PB, AG, and OB wrote the manuscript. All authors read and approved the manuscript.

## Conflict of Interest

The authors declare that the research was conducted in the absence of any commercial or financial relationships that could be construed as a potential conflict of interest.

## Publisher’s Note

All claims expressed in this article are solely those of the authors and do not necessarily represent those of their affiliated organizations, or those of the publisher, the editors and the reviewers. Any product that may be evaluated in this article, or claim that may be made by its manufacturer, is not guaranteed or endorsed by the publisher.

## References

[B1] Aby K. Antony R. Eichholz M. Srinivasan R. Li Y. (2021). Enhanced pro-BDNF-p75NTR pathway activity in denervated skeletal muscle. *Life Sci.* 286:120067. 10.1016/J.LFS.2021.120067 34678261 PMC8595791

[B2] Anastasia A. Deinhardt K. Chao M. V. Will N. E. Irmady K. Lee F. S. (2013). Val66Met polymorphism of BDNF alters prodomain structure to induce neuronal growth cone retraction. *Nat. Commun.* 4:2490. 10.1038/ncomms3490 24048383 PMC3820160

[B3] Argentieri T. M. Aiken S. P. Laxminarayan S. McArdle J. J. (1992). Characteristics of synaptic transmission in reinnervating rat skeletal muscle. *Pflügers Arch.* 421 256–261. 10.1007/BF00374835 1326747

[B4] Assaife-Lopes N. Sousa V. C. Pereira D. B. Ribeiro J. A. Sebastião A. M. (2014). Regulation of TrkB receptor translocation to lipid rafts by adenosine A2A receptors and its functional implications for BDNF-induced regulation of synaptic plasticity. *Purinergic Signal.* 10 251–267. 10.1007/S11302-013-9383-2 24271058 PMC4040169

[B5] Balezina O. P. Bogacheva P. O. Orlova T. Y. (2007). Effect of L-type calcium channel blockers on activity of newly formed synapses in mice. *Bull. Exp. Biol. Med.* 143 171–174. 10.1007/S10517-007-0041-Y 17970192

[B6] Bogatcheva P. O. Balezina O. P. (2013). Multidirectional effects of calmodulin kinase ii on transmitter release in mature and newly formed mouse motor synapses. *Bull. Exp. Biol. Med.* 154 316–319. 10.1007/S10517-013-1940-8 23484190

[B7] Brooke R. E. Moores T. S. Morris N. P. Parson S. H. Deuchars J. (2004). Kv3 voltage-gated potassium channels regulate neurotransmitter release from mouse motor nerve terminals. *Eur. J. Neurosci.* 20 3313–3321. 10.1111/J.1460-9568.2004.03730.X 15610163

[B8] Cao X. H. Chen S. R. Li L. Pan H. L. (2012). Nerve injury increases brain-derived neurotrophic factor levels to suppress BK channel activity in primary sensory neurons. *J. Neurochem.* 121 944–953. 10.1111/J.1471-4159.2012.07736.X 22428625 PMC3371106

[B9] Castrén E. Antila H. (2017). Neuronal plasticity and neurotrophic factors in drug responses. *Mol. Psychiatry* 228 1085–1095. 10.1038/mp.2017.61 28397840 PMC5510719

[B10] Costa R. O. Perestrelo T. Almeida R. D. (2017). PROneurotrophins and CONSequences. *Mol. Neurobiol.* 554 2934–2951. 10.1007/S12035-017-0505-7 28456935

[B11] Coulson E. J. May L. M. Osborne S. L. Reid K. Underwood C. K. Meunier F. A. (2008). p75 Neurotrophin receptor mediates neuronal cell death by activating GIRK channels through phosphatidylinositol 4,5-bisphosphate. *J. Neurosci.* 28 315–324. 10.1523/JNEUROSCI.2699-07.2008 18171948 PMC6671158

[B12] Dascal N. (1997). Signalling via the G Protein-Activated K+ Channels. *Cell. Signal.* 9 551–573. 10.1016/S0898-6568(97)00095-8 9429760

[B13] De Vincenti A. P. Ríos A. S. Paratcha G. Ledda F. (2019). Mechanisms that modulate and diversify BDNF functions: Implications for hippocampal synaptic plasticity. *Front. Cell. Neurosci.* 13:135. 10.3389/FNCEL.2019.00135 31024262 PMC6465932

[B14] Dieni S. Matsumoto T. Dekkers M. Rauskolb S. Ionescu M. S. Deogracias R. (2012). BDNF and its pro-peptide are stored in presynaptic dense core vesicles in brain neurons. *J. Cell Biol.* 196 775–788. 10.1083/JCB.201201038 22412021 PMC3308691

[B15] Dodson P. D. Forsythe I. D. (2004). Presynaptic K+ channels: electrifying regulators of synaptic terminal excitability. *Trends Neurosci.* 27 210–217. 10.1016/J.TINS.2004.02.012 15046880

[B16] Dvoriantchikova G. Ivanov D. Barakat D. Grinberg A. Wen R. Slepak V. Z. (2012). Genetic ablation of pannexin1 protects retinal neurons from ischemic injury. *PLoS One* 7:e31991. 10.1371/JOURNAL.PONE.0031991 22384122 PMC3285635

[B17] Fernández-Fernández D. Lamas J. A. (2021). Metabotropic modulation of potassium channels during synaptic plasticity. *Neuroscience* 456 4–16. 10.1016/J.NEUROSCIENCE.2020.02.025 32114098

[B18] Flink M. T. Atchison W. D. Atchison B. (2003). Iberiotoxin-Induced block of Ca2+-Activated K+ Channels Induces dihydropyridine sensitivity of ACh release from mammalian motor nerve terminals. *J. Pharmacol. Exp. Ther.* 305 646–652. 10.1124/JPET.102.046102 12606686

[B19] Garcia N. Santafe M. M. Tomàs M. Lanuza M. A. Besalduch N. Tomàs J. (2010a). Involvement of brain-derived neurotrophic factor (BDNF) in the functional elimination of synaptic contacts at polyinnervated neuromuscular synapses during development. *J. Neurosci. Res.* 88 1406–1419. 10.1002/JNR.22320 20029969

[B20] Garcia N. Tomàs M. Santafe M. M. Lanuza M. A. Besalduch N. Tomàs J. (2010b). Localization of brain-derived neurotrophic factor, neurotrophin-4, tropomyosin-related kinase b receptor, and p75NTR receptor by high-resolution immunohistochemistry on the adult mouse neuromuscular junction. *J. Peripher. Nerv. Syst.* 15 40–49. 10.1111/J.1529-8027.2010.00250.X 20433604

[B21] Garcia N. Tomàs M. Santafe M. M. Lanuza M. A. Besalduch N. Tomàs J. (2011). Blocking p75NTR receptors alters polyinnervationz of neuromuscular synapses during development. *J. Neurosci. Res.* 89 1331–1341. 10.1002/JNR.22620 21674565

[B22] Gaydukov A. Bogacheva P. Tarasova E. Molchanova A. Miteva A. Pravdivceva E. (2019). Regulation of acetylcholine quantal release by coupled thrombin/BDNF signaling in mouse motor synapses. *Cells* 8:762. 10.3390/CELLS8070762 31336670 PMC6678150

[B23] Gaydukov A. E. Akutin I. A. Bogacheva P. O. Balezina O. P. (2018). Changes in the parameters of quantal acetylcholine release after activation of PAR1-type thrombin receptors at the mouse neuromuscular junctions. *Biochem. (Mosc.) Suppl. A Membr. Cell Biol.* 12 33–42. 10.1134/S1990747818010063

[B24] Gaydukov A. E. Bogacheva P. O. Balezina O. P. (2016). Calcitonin gene-related peptide increases acetylcholine quantal size in neuromuscular junctions of mice. *Neurosci. Lett.* 628 17–23. 10.1016/J.NEULET.2016.06.014 27288020

[B25] Gaydukov A. E. Bogacheva P. O. Tarasova E. O. Balezina O. P. (2014). The mechanism of choline-mediated inhibition of acetylcholine release in mouse motor synapses. *Acta Naturae* 6 110–115. PMC4273098 25558401

[B26] Gaydukov A. E. Melnikova S. N. Balezina O. P. (2009). Facilitation of acetylcholine secretion in mouse motor synapses caused by calcium release from depots upon activation of l-type calcium channels. *Bull. Exp. Biol. Med.* 148 163–166. 10.1007/S10517-009-0678-9 20027318

[B27] Gray K. Ellis V. (2008). Activation of pro-BDNF by the pericellular serine protease plasmin. *FEBS Lett.* 582 907–910. 10.1016/J.FEBSLET.2008.02.026 18291105

[B28] Guarracino J. F. Cinalli A. R. Fernández V. Roquel L. I. Losavio A. S. (2016). P2Y13 receptors mediate presynaptic inhibition of acetylcholine release induced by adenine nucleotides at the mouse neuromuscular junction. *Neuroscience* 326 31–44. 10.1016/J.NEUROSCIENCE.2016.03.066 27058149

[B29] Guiler W. Koehler A. Boykin C. Lu Q. (2021). Pharmacological modulators of small GTPases of rho family in neurodegenerative diseases. *Front. Cell. Neurosci.* 15:150. 10.3389/FNCEL.2021.661612/BIBTEX PMC8149604 34054432

[B30] Haddad Y. Adam V. Heger Z. (2017). Trk receptors and neurotrophin cross-interactions: new perspectives toward manipulating therapeutic side-effects. *Front. Mol. Neurosci.* 10:130. 10.3389/FNMOL.2017.00130 28515680 PMC5414483

[B31] Hamilton B. R. Smith D. O. (1991). Autoreceptor-mediated purinergic and cholinergic inhibition of motor nerve terminal calcium currents in the rat. *J. Physiol.* 432 327–341. 10.1113/JPHYSIOL.1991.SP018387 1653322 PMC1181328

[B32] Hibino H. Inanobe A. Furutani K. Murakami S. Findlay I. Kurachi Y. (2010). Inwardly rectifying potassium channels: Their structure, function, and physiological roles. *Physiol. Rev.* 90 291–366. 10.1152/PHYSREV.00021.2009 20086079

[B33] Hill E. Hickman C. Diez R. Wall M. (2020). Role of A1 receptor-activated GIRK channels in the suppression of hippocampal seizure activity. *Neuropharmacology* 164:107904. 10.1016/J.NEUROPHARM.2019.107904 31812775

[B34] Huang E. J. Reichardt L. F. (2003). Neurotrophins: roles in neuronal development and function. *Annu. Rev. Neuro.* 24 677–736. 10.1146/ANNUREV.NEURO.24.1.677 11520916 PMC2758233

[B35] Hurtado E. Cilleros V. Nadal L. Simó A. Obis T. Garcia N. (2017). Muscle contraction regulates bdnf/trkb signaling to modulate synaptic function through presynaptic cPKCα and cPKCβi. *Front. Mol. Neurosci.* 10:147. 10.3389/FNMOL.2017.00147 28572757 PMC5436293

[B36] Je H. S. Yang F. Ji Y. Nagappan G. Hempstead B. L. Lu B. (2012). Role of pro-brain-derived neurotrophic factor (proBDNF) to mature BDNF conversion in activity-dependent competition at developing neuromuscular synapses. *Proc. Natl. Acad. Sci. U.S.A.* 109 15924–15929. 10.1073/PNAS.1207767109 23019376 PMC3465384

[B37] Je H. S. Yang F. Ji Y. Potluri S. Fu X. Q. Luo Z. G. (2013). ProBDNF and mature BDNF as punishment and reward signals for synapse elimination at mouse neuromuscular junctions. *J. Neurosci.* 33 9957–9962. 10.1523/JNEUROSCI.0163-13.2013 23761891 PMC3682390

[B38] Jeremic D. Sanchez-Rodriguez I. Jimenez-Diaz L. Navarro-Lopez J. D. (2021). Therapeutic potential of targeting G protein-gated inwardly rectifying potassium (GIRK) channels in the central nervous system. *Pharmacol. Ther.* 223:107808. 10.1016/J.PHARMTHERA.2021.107808 33476640

[B39] Kilpatrick L. E. Hill S. J. (2021). Transactivation of G protein-coupled receptors (GPCRs) and receptor tyrosine kinases (RTKs): recent insights using luminescence and fluorescence technologies. *Curr. Opin. Endocr. Metab. Res* 16 102–112. 10.1016/J.COEMR.2020.10.003 33748531 PMC7960640

[B40] Kim C. S. Johnston D. (2015). A1 adenosine receptor-mediated GIRK channels contribute to the resting conductance of CA1 neurons in the dorsal hippocampus. *J. Neurophysiol.* 113 2511–2523. 10.1152/JN.00951.2014 25652929 PMC4416607

[B41] Kojima M. Matsui K. Mizui T. (2019). BDNF pro-peptide: physiological mechanisms and implications for depression. *Cell. Tissue Res.* 377 73–79. 10.1007/S00441-019-03034-6 31076872

[B42] Koshimizu H. Kiyosue K. Hara T. Hazama S. Suzuki S. Uegaki K. (2009). Multiple functions of precursor BDNF to CNS neurons: negative regulation of neurite growth, spine formation and cell survival. *Mol. Brain* 2:27. 10.1186/1756-6606-2-27 19674479 PMC2743674

[B43] Lu B. (2003). Pro-Region of neurotrophins: role in synaptic modulation. *Neuron* 39 735–738. 10.1016/S0896-6273(03)00538-5 12948441

[B44] Luján R. Aguado C. (2015). Localization and targeting of GIRK channels in mammalian central neurons. *Int. Rev. Neurobiol.* 123 161–200. 10.1016/BS.IRN.2015.05.009 26422985

[B45] Lupica C. R. Bell J. A. Hoffman A. F. Watson P. L. (2001). Contribution of the hyperpolarization-activated current (Ih) to membrane potential and GABA release in hippocampal interneurons. *J. Neurophysiol.* 86 261–268. 10.1152/JN.2001.86.1.261 11431507

[B46] Lüscher C. Slesinger P. A. (2010). Emerging roles for G protein-gated inwardly rectifying potassium (GIRK) channels in health and disease. *Nat. Rev. Neurosci.* 11 301–315. 10.1038/NRN2834 20389305 PMC3052907

[B47] Malik S. C. Sozmen E. G. Baeza-Raja B. Le Moan N. Akassoglou K. Schachtrup C. (2021). In vivo functions of p75NTR: challenges and opportunities for an emerging therapeutic target. *Trends Pharmacol. Sci.* 42 772–788. 10.1016/J.TIPS.2021.06.006 34334250 PMC8502531

[B48] Matsumoto T. Rauskolb S. Polack M. Klose J. Kolbeck R. Korte M. (2008). Biosynthesis and processing of endogenous BDNF: CNS neurons store and secrete BDNF, not pro-BDNF. *Nat. Neurosci.* 112 131–133. 10.1038/nn2038 18204444

[B49] May L. M. Anggono V. Gooch H. M. Jang S. E. Matusica D. Kerbler G. M. (2017). G-Protein-Coupled inwardly rectifying potassium (GIRK) channel activation by the p75 neurotrophin receptor is required for amyloid β toxicity. *Front. Neurosci.* 11:455. 10.3389/FNINS.2017.00455 28848381 PMC5550722

[B50] McArdle J. J. Albuquerque E. X. (1973). A Study of the reinnervation of fast and slow mammalian muscles. *J. Gen. Physiol.* 61 1–23. 10.1085/JGP.61.1.1 4683095 PMC2203463

[B51] McLachlan E. M. Martin A. R. (1981). Non-linear summation of end-plate potentials in the frog and mouse. *J. Physiol.* 311 307–324. 10.1113/JPHYSIOL.1981.SP013586 6267255 PMC1275411

[B52] Meeker R. Williams K. (2014). Dynamic Nature of the p75 neurotrophin receptor in response to injury and disease. *J. Neuroimmune Pharmacol.* 9 615–628. 10.1007/S11481-014-9566-9 25239528 PMC4243516

[B53] Meir A. Ginsburg S. Butkevich A. Kachalsky S. G. Kaiserman I. Ahdut R. (1999). Ion channels in presynaptic nerve terminals and control of transmitter release. *Physiol. Rev.* 79 1019–1088. 10.1152/PHYSREV.1999.79.3.1019 10390521

[B54] Meneses D. Mateos V. Islas G. Barral J. (2015). G-protein-coupled inward rectifier potassium channels involved in corticostriatal presynaptic modulation. *Synapse* 69 446–452. 10.1002/SYN.21833 26173917

[B55] Miteva A. Gaydukov A. Balezina O. (2020). Interaction between calcium chelators and the activity of P2X7 receptors in mouse motor synapses. *Int. J. Mol. Sci.* 21:2034. 10.3390/IJMS21062034 32188153 PMC7139400

[B56] Miteva A. S. Gaydukov A. E. Shestopalov V. I. Balezina O. P. (2017). The role of pannexin 1 in the purinergic regulation of synaptic transmission in mouse motor synapses. *Biochem. (Mosc.) Suppl. A Membr. Cell Biol.* 114 311–320. 10.1134/S1990747817040067

[B57] Miteva A. S. Gaydukov A. E. Shestopalov V. I. Balezina O. P. (2018). Mechanism of P2X7 receptor-dependent enhancement of neuromuscular transmission in pannexin 1 knockout mice. *Purinergic Signal.* 14 459–469. 10.1007/S11302-018-9630-7 30362043 PMC6298918

[B58] Mizoguchi H. Nakade J. Tachibana M. Ibi D. Someya E. Koike H. (2011). Matrix metalloproteinase-9 contributes to kindled seizure development in pentylenetetrazole-treated mice by converting pro-BDNF to mature BDNF in the hippocampus. *J. Neurosci.* 31 12963–12971. 10.1523/JNEUROSCI.3118-11.2011 21900575 PMC6623408

[B59] Mizui T. Ishikawa Y. Kumanogoh H. Kojima M. (2016). Neurobiological actions by three distinct subtypes of brain-derived neurotrophic factor: multi-ligand model of growth factor signaling. *Pharmacol. Res.* 105 93–98. 10.1016/J.PHRS.2015.12.019 26747403

[B60] Nagappan G. Zaitsev E. Senatorov V. V. Yang J. Hempstead B. L. Lu B. (2009). Control of extracellular cleavage of ProBDNF by high frequency neuronal activity. *Proc. Natl. Acad. Sci. U.S.A.* 106 1267–1272. 10.1073/PNAS.0807322106 19147841 PMC2633536

[B61] Nykjaer A. Willnow T. E. Petersen C. M. (2005). p75NTR – live or let die. *Curr. Opin. Neurobiol.* 15 49–57. 10.1016/J.CONB.2005.01.004 15721744

[B62] Pang P. T. Teng H. K. Zaitsev E. Woo N. T. Sakata K. Zhen S. (2004). Cleavage of proBDNF by tPA/plasmin is essential for long-term hippocampal plasticity. *Science* 306 487–491. 10.1126/SCIENCE.1100135 15486301

[B63] Park H. Poo M. M. (2012). Neurotrophin regulation of neural circuit development and function. *Nat. Rev. Neurosci.* 141 7–23. 10.1038/nrn3379 23254191

[B64] Perissinotti P. P. Uchitel O. D. (2010). Adenosine drives recycled vesicles to a slow-release pool at the mouse neuromuscular junction. *Eur. J. Neurosci.* 32 985–996. 10.1111/J.1460-9568.2010.07332.X 20726887

[B65] Phillips C. Baktir M. A. Srivatsan M. Salehi A. (2014). Neuroprotective effects of physical activity on the brain: a closer look at trophic factor signaling. Front. *Cell. Neurosci.* 8:170. 10.3389/FNCEL.2014.00170 24999318 PMC4064707

[B66] Ponce A. Bueno E. Kentros C. Vega-Saenz De Miera E. Chow A. Hillman D. (1996). G-protein-gated inward rectifier K+ channel proteins (GIRK1) are present in the soma and dendrites as well as in nerve terminals of specific neurons in the brain. *J. Neurosci.* 16 1990–2001. 10.1523/JNEUROSCI.16-06-01990.1996 8604043 PMC6578514

[B67] Pousinha P. A. José Diogenes M. Alexandre Ribeiro J. Sebastião A. M. (2006). Triggering of BDNF facilitatory action on neuromuscular transmission by adenosine A2A receptors. *Neurosci. Lett.* 404 143–147. 10.1016/J.NEULET.2006.05.036 16790314

[B68] Ribeiro J. A. Cunha R. A. Correia-de-Sa P. Sebastiao A. M. (1996). Purinergic regulation of acetylcholine release. *Prog. Brain Res.* 109 231–241. 10.1016/s0079-6123(08)62107-x 9009712

[B69] Riffault B. Medina I. Dumon C. Thalman C. Ferrand N. Friedel P. (2014). Pro-Brain-Derived neurotrophic factor inhibits GABAergic neurotransmission by activating endocytosis and repression of GABAA receptors. *J. Neurosci.* 34 13516–13534. 10.1523/JNEUROSCI.2069-14.2014 25274828 PMC6608319

[B70] Rogalski S. L. Appleyard S. M. Pattillo A. Terman G. W. Chavkin C. (2000). TrkB Activation by Brain-derived neurotrophic factor inhibits the g protein-gated inward rectifier Kir3 by tyrosine phosphorylation of the channel. *J. Biol. Chem.* 275 25082–25088. 10.1074/JBC.M000183200 10833508 PMC1276699

[B71] Roncarati R. Di Chio M. Sava A. Terstappen G. C. Fumagalli G. (2001). Presynaptic localization of the small conductance calcium-activated potassium channel SK3 at the neuromuscular junction. *Neuroscience* 104 253–262. 10.1016/S0306-4522(01)00066-5 11311547

[B72] Sun Q. Q. Akk G. Huguenard J. R. Prince D. A. (2001). Differential regulation of GABA release and neuronal excitability mediated by neuropeptide Y1 and Y2 receptors in rat thalamic neurons. *J. Physiol.* 531 81–94. 10.1111/J.1469-7793.2001.0081J.X 11179393 PMC2278458

[B73] Tomàs J. Garcia N. Lanuza M. A. Santafé M. M. Tomàs M. Nadal L. (2017). Presynaptic membrane receptors modulate ACh release, axonal competition and synapse elimination during neuromuscular junction development. *Front. Mol. Neurosci.* 10:132. 10.3389/FNMOL.2017.00132 28559796 PMC5432534

[B74] Vansaun M. Herrera A. A. Werle M. J. (2003). Structural alterations at the neuromuscular junctions of matrix metalloproteinase 3 null mutant mice. *J. Neurocytol.* 329 1129–1142. 10.1023/B:NEUR.0000021907.68461.9C 15044844

[B75] Vázquez-Vázquez H. Gonzalez-Sandoval C. Vega A. V. Arias-Montaño J. A. Barral J. (2020). Histamine H3 receptor activation modulates glutamate release in the corticostriatal synapse by acting at CaV2.1 (P/Q-Type) calcium channels and GIRK (KIR3) potassium channels. *Cell. Mol. Neurobiol.* 42 817–828. 10.1007/S10571-020-00980-6 33068216 PMC11441178

[B76] Wang C. S. Kavalali E. T. Monteggia L. M. (2022). BDNF signaling in context: from synaptic regulation to psychiatric disorders. *Cell* 185 62–76. 10.1016/J.CELL.2021.12.003 34963057 PMC8741740

[B77] Wang M. Xie Y. Qin D. (2021). Proteolytic cleavage of proBDNF to mBDNF in neuropsychiatric and neurodegenerative diseases. *Brain Res. Bull* 166 172–184. 10.1016/j.brainresbull.2020.11.005 33202257

[B78] Wang X. Burke S. R. A. Talmadge R. J. Voss A. A. Rich M. M. (2020). Depressed neuromuscular transmission causes weakness in mice lacking BK potassium channels. *J. Gen. Physiol.* 152:e201912526. 10.1085/JGP.201912526/151617 32243496 PMC7201880

[B79] Yang F. Je H. S. Ji Y. Nagappan G. Hempstead B. Lu B. (2009). Pro-BDNF–induced synaptic depression and retraction at developing neuromuscular synapses. *J. Cell Biol.* 185 727–741. 10.1083/JCB.200811147 19451278 PMC2711569

[B80] Yum D. S. Cho J. H. Choi I. S. Nakamura M. Lee J. J. Lee M. G. (2008). Adenosine A1 receptors inhibit GABAergic transmission in rat tuberomammillary nucleus neurons. *J. Neurochem.* 106 361–371. 10.1111/J.1471-4159.2008.05400.X 18397365

[B81] Zanin J. P. Unsain N. Anastasia A. (2017). Growth factors and hormones pro-peptides: the unexpected adventures of the BDNF prodomain. *J. Neurochem.* 141 330–340. 10.1111/JNC.13993 28218971

